# Supplementary material for: Are carbon emissions trading and green financial instruments synergistic? -Comprehensive quantitative research based on content analysis
Source: PLoS One. 2024 Mar 7;19(3):e0298601. doi: 10.1371/journal.pone.0298601 (PMC10919601; doi:10.1371/journal.pone.0298601)
Supplement: S1 Data — (ZIP) [file pone.0298601.s001.zip › Data/地级市Z_F工作报告.zip!/║■▒▒/╢⌡╓▌╩╨/└┤╫╘╣1⁄2╓┌║┼í╛┬φ┐╦╩2╛▌═°í┐_2011.docx]

各位代表：

　　现在，我代表市人民政府向大会作政府工作报告，请连同《鄂州市国民经济和社会发展第十二个五年规划纲要 (草案)》一并审议，并请各位市政协委员和列席人员提出意见。

　　一、2010年工作回顾

　　2010年，我市经济社会发展取得显著成就。一年来，面对复杂多变的国内外经济形势和影响严重的自然灾害，深入贯彻落实科学发展观，紧紧依靠全市广大干部群众，着力转方式、调结构，经济快速增长，民生持续改善，社会和谐稳定。本届人大四次会议确定的2010年主要预期目标均超额完成。

　　——有效应对挑战，经济发展跨上新台阶。经济总量不断壮大。有效应对后危机时期挑战和十年一遇的暴雨洪涝灾害冲击，经济发展实现新跨越。全年实现地区生产总值395.3亿元，同比增长15.3%；财政总收入31.5亿元，增长18.5%；地方一般预算收入15.7亿元，增长20.3%。投资规模不断扩大。完成全社会固定资产投资298.6亿元，增长35.4%。重点项目进展顺利。进一步加大重点项目督办协调服务力度，年初确定的115个市级重点项目全部完成年度投资计划。鄂州电厂二期、武大有机硅、银龙管业等35个项目试生产或投产；航宇国际商贸城、磨石山铁矿、金刚石科技园等15个项目完成年度投资计划；华顶机电工业园、华工科技等58个项目开工建设，比2009年增加11个。不断加大向上争取资金力度，共争取国家和省发改委投资项目370个，争取到位资金7.13亿元，带动项目投资近40亿元。区级实力不断增强。区级经济发展提速，地区生产总值等主要经济指标的总量及增幅均高于市直。在全省县域经济综合考评中，鄂城区居前20位，华容区居前30位，梁子湖区进位明显。葛店开发区与东湖高新区对接融合力度加大，鄂州开发区建设步伐加快，花湖工贸新城初具规模，鄂城新区完成了分区概念性规划，红莲湖旅游度假区和梧桐湖新区对接进展顺利。

　　——实施结构调整，发展方式得到新转变。着力推进新型工业化。大力推进千亿元产业发展计划，冶金、装备制造、建材、能源、医药等支柱产业优势凸显，形成了近40个门类的工业体系。工业经济在优化结构中加快发展，实现规模以上工业增加值205.44亿元，增长26.1%，增幅居武汉城市圈第1位。企业结构提档升级，中省企业核心竞争力、带动作用明显增强；实施中小企业成长工程，净增规模工业企业50家，新增销售收入过亿元企业25家；湖北大通运业股份有限公司、顾地科技股份有限公司上市工作取得重大进展。推进质量兴市和名牌发展战略，“鄂州武昌鱼”被认定为中国驰名商标、湖北名牌产品。大力发展高新技术产业，高新技术企业达到74家，其中国家级高新技术企业14家，省级创新试点企业10家，完成增加值47.9亿元，增长29.6%。产业集群快速扩张，形成了10大成长性产业集群，金刚石刀具、生物医药、钢铁深加工3大产业集群发展成为省重点成长型产业集群。着力推进服务业跨越发展。出台了《加快服务业跨越发展的实施意见》，服务业实现增加值112.49亿元，增长9%。三江港区、主城区和花湖工贸新城三大物流基地建设取得重大进展。旅游业发展势头强劲，制定了《关于加快推进旅游业成为战略性支柱产业的意见》，实现总收入16.23亿元，增长23.6%，第十一届梁子湖捕鱼旅游节成功举办。金融支持经济发展力度明显加大，成功引进中信银行在我市开设分支机构，全市各项存款余额达到256.6亿元，增长26.7%；各项贷款余额达到141.7亿元，增长42.3%，分别高于全市GDP增幅、全省平均增幅、去年同期增幅27、20.8和15.3个百分点；实现保费收入6.2亿元，增长27.8%。文化、信息、社区服务等新兴服务业发展加快。着力推进消费拉动经济增长。积极实施“家电下乡”、“汽车、摩托车下乡”等一系列鼓励消费政策，消费环境进一步优化，城乡市场进一步繁荣，消费结构加快升级，完成社会消费品零售总额135.6亿元，增长20.1%。着力推进节能减排。大力淘汰落后产能，积极实施重点减排工程。成立了鄂州市节能监测中心，圆满完成省政府下达的小纺织、小印染落后产能淘汰任务，鄂州电厂二期3号、4号机组脱硫设施投入运行，鄂钢公司焦化酚氰废水改造工程建成投入运行，鄂钢公司90㎡烧结机烟气脱硫工程竣工并通过环保部验收，我市被列为全国第四批节水型社会试点城市。全市单位生产总值综合能耗、化学需氧量和二氧化硫排放量分别下降0.8%、0.5%和2.5%。

　　——突出统筹发展，城乡一体化进入新阶段。城乡一体化试点向纵深推进。积极推进特色镇和新社区建设，一批商品集散型、工业主导型、特色旅游型城镇初现雏形；开工建设43个新社区，28个新社区初具规模，社区服务中心“1+8”管理服务模式基本形成。扎实开展长港示范带建设，取得明显成效。加快推进均衡公共服务，30个方面的城乡公共服务稳步均衡。现代农业加快发展。农业增加值实现51.45亿元，增长5.2%。农民人均纯收入达到6645元，增长16.2%。鄂州农产品加工园被列为全省重点加工园区。认真落实国家粮食最低收购价格政策，粮食订单经营成效明显。水产、蔬菜、畜禽、林果等四大支柱产业增加值达到农业总量的80%。农业市级重点龙头企业达到44个，省级重点龙头企业达到7个，形成了水产加工、畜禽加工、花卉苗木等7条产业链。全市农业产业化经营组织达597个，农业专业合作社达183个，新办农业综合服务社92家。农村生产生活条件加快改善。完成12万亩国土整理项目建设。完成3.7万亩造林工程。实施了洋澜湖水域环境全面整治、河流泵站改造、水库除险加固等重点水利工程建设。完成224公里农村公路建设和8座县、乡、村公路旧桥整险加固工程。对农民购置农机实施财政直补，综合机械化水平由52.6%提高到56.8%。气象灾害预报预警深入开展，为农业服务能力明显提升。自来水主管网实现乡镇全覆盖，65万农村人口喝上自来水，鄂城区成为全国农村安全饮水工程示范区。广播电视综合覆盖率达95%以上，有线电视通村率达到90%。集镇管道燃气使用率达到50%，家用沼气池达到2.2万口。深化“清洁乡村、美化家园”活动，推进农村环境综合连片整治，农村人居环境明显改善。扶贫工作取得显著成效，市老促会被评为全国先进单位。

　　成功承办了全省城乡一体化工作会议，提升了鄂州在全省乃至全国的地位和形象。中共中央政治局委员、中组部部长李源潮来鄂州考察，对我市城乡一体化工作给予了较高评价。

　　——增强发展活力，改革开放取得新突破。政府机构改革顺利完成。平稳推进市区两级政府机构改革，着力建立精简高效的大部门制。全面提升机构编制实名制管理工作水平，全国机构编制实名管理工作经验交流现场会在我市召开。重点领域改革深入推进。全面启动医药卫生体制改革，我市被列为全国公立医院改革试点城市，改革试点工作取得了重大突破，得到中央政治局常委、国务院副总理李克强的高度肯定。城管综合执法、文化综合执法等改革稳步推进。农村信用社产权制度改革正式启动。“两型”社会示范区建设取得重大进展。《武汉城市圈“两型”社会建设鄂州示范区改革发展纲要（2010-2020）》通过专家论证，相关方案和行动计划正抓紧编制完善。开放型经济水平不断提高。出台了《关于加快发展开放型经济的若干意见》，举办了一系列大型经贸洽谈活动，新签约项目150个，其中亿元以上项目76个，创历史最好水平。完成招商引资总额131.98亿元，增长51.87%；实际利用外资1.24亿美元，增长10%；外贸出口1.29亿美元，增长75.8%。

　　——提升承载能力，城市建设展现新形象。交通建设超常发展。完成交通投资15亿元，同比翻两番。汉鄂快速通道、大广高速鄂州段建设进展顺利，鄂黄高速开工建设，葛湖一级公路路基主体工程完成，鄂燕黄路、铁贺线和106国道大修改造顺利完成，樊口大桥连接线和樊吉线相继建成通车；武鄂黄（黄）两条城际铁路、鄂州火车站改造进展顺利，三江港货运站及港区铁路工程开工建设；完成港口建设投资1.39亿元，武汉新港三江港区建设进展顺利，鄂钢工业港项目开工，三江港区武钢集团泊位工程获交通运输部批复，五丈港多功能码头岸线使用通过评审，湖北星丰金属资源利用有限公司废金属加工项目及码头工程项目已通过国家发改委审批。城市建设全面提速。积极拓宽融资渠道，十大基础设施建设工程全面推进，共完成投资7.5亿元。城市东西中轴线、南北中轴线、三国吴都风光带、洋澜湖生态修复治理等重点工程建设有序推进，寿昌大道中段、寒溪路、樊蒲路、学府路进展顺利，樊口污水处理厂建成，完成30条背街小巷改造，城市功能进一步完善，城市骨架进一步拉开。城市管理明显加强。实施《城区“门前四包、庭院达标”检查考核奖惩制度》，免费开放79座直管公厕，开辟14处市容疏导场所，完成4座大中型城市电视。开展了城乡一体化垃圾收集清运系统试点，全市污水处理率、生活垃圾处理率分别达65.42%、82.6%。开展了市容市貌十大整治、饮食摊点集中整治等专项执法行动，城市管理难题得到有效化解，市容市貌和环境秩序进一步改善。推进城市管理重心下移，街道办事处的职能进一步转变，城市建设、管理作用显著增强。“四城联创”扎实推进，省级卫生城顺利通过复检。

　　——坚持共建共享，民生和社会事业有了新改善。努力为群众办实事。筹集资金11.03亿元，市政府年初承诺的“十件实事”全面兑现。全力推进就业创业工作。大力实施市场主体增量行动，制定了《关于进一步加强市场主体发展促进经济平稳较快发展的意见》，全市新登记各类市场主体5985户。发放小额担保贷款5889万元，扶持民营企业和个体工商户共2311家。实施就业援助行动，开发公益性岗位1800个。城镇新增就业2.1万人，城镇登记失业率为4.1%，低于全省目标控制数。城镇居民人均可支配收入达到14788元，增长10.3%。不断完善社会保障体系。全市五项基本社会保险新增扩面3.1万人次，征收社会保险费6.9亿元。城乡医疗保险一体化加速推进，全市综合参保率达93%，为116万人次支付医疗保险基金2.37亿元，成为全省唯一实现医疗保险市级统筹、门诊统筹、城乡统筹的地级市。梁子湖区新农保试点工作进展顺利，参保人数超过10万人，2万名农村老人享受了养老金。启动了华容区、葛店开发区新农保工作。调整最低工资标准，提高企业退休人员养老金标准，切实保障中低收入人群基本生活。加大社会救助力度，推进低保工作规范化管理，城乡低保保障人口4.3万人，发放低保资金6000万元，城乡低保标准提高20%以上；取消病种限制，初步实现医疗救助全覆盖。改善住房困难家庭住房条件，建设廉租住房1154套，发放住房租赁补贴420万元。发放住房公积金个人贷款3.3亿元，使2027户中低收入家庭圆了住房梦。扎实做好防汛抗灾工作。面对1998年以来最强降雨的袭击，精心部署，科学调度，统筹安排，坚持一手抓防汛抗灾，一手抓生产自救，全力做好应急管理、生活救助、物资供给和抗灾复产等工作，转移安置受灾群众13844人，圆满完成755户倒房重建任务，洪涝灾害的影响降到了最低程度，防汛救灾取得了全面胜利。梁子湖区被授予“全国防汛抗旱先进集体”荣誉称号。大力发展社会事业。坚持优先发展教育，大力实施中小学校舍安全工程，基本消灭D级危房，重建校舍61栋、新改扩建面积2.2万平方米，规范办学年和教学大比武活动成效明显，建立了义务教育经费保障新机制，鄂州职业大学跻身全国百所骨干高职院校行列。科技事业快速发展，获得省级科技奖9项，取得省级科技成果12项，专利申请量突破700件，通过全国知识产权试点城市考核验收。促进文化大发展大繁荣，在全省率先实现综合文化站乡镇全覆盖和农民体育健身工程、农家书屋村级全覆盖，率先启动农村有线电视电子书屋工程，数字电视整体转换工作稳步推进，鄂州籍运动员首次在亚运会上获得金牌，梁子湖区穿花龙舟获第二届中国龙舟文化节展演赛金奖，“周周乐”成为全国、全省文化知名品牌，鄂州雕花剪纸和牌子锣分别入选联合国、国家级非物质文化遗产名录。加快建立和完善覆盖城乡居民的基本医疗卫生制度，加强基本医疗服务体系建设，完成了80个区医院、乡镇卫生院、社区卫生服务站和村卫生室的新建和改扩建任务，相继在鄂城区、梁子湖区、葛店开发区实施了基本药物制度改革试点，公共卫生服务均衡化在城乡全面铺开。积极发展社会养老服务事业，城市福利中心托养护理工程完工，5所农村福利院达到省级以上模范标准。采取多种调控措施，保持物价基本稳定。统计工作进一步加强，扎实做好第六次人口普查工作。人口计生工作取得新的进步，取得全省综合考核和部门线考核两个进步奖。社区建设和村民自治工作深入开展。农村基层组织建设得到加强，村级集体经济加快发展。推进残疾人事业发展，制定出台了《关于促进残疾人事业发展的意见》，着力推进残疾人社区康复服务，被授予“全国残疾人社区康复示范市”。国防教育和国防后备力量建设不断加强，军政军民团结更加巩固，通过省级“双拥模范城”考核验收。加强人民防空基础设施建设，人防应急综合能力得到提高。“五五”普法顺利通过检查验收。社会治安综合治理得到加强，连续五年实现命案全破，群众安全感不断提升，荣获全省社会治安综合治理优胜单位。不断拓宽社情民意诉求渠道，大力开展大宣讲、大排查、大接访、大调处活动，促进了社会和谐稳定。严格落实安全生产责任制，建立重大危险源监控网络，加大对重点行业安全隐患的排查整改力度，严格查处安全生产事故，突出做好消防安全工作，提高应急救援能力。安全生产四项指标连续五年下降，是全省唯一没有发生较大安全事故的市州。加大对食品药品安全监管力度，加强烟草等专卖市场打假力度，努力维护市场经济秩序。对口支援四川抗震救灾工作任务全面完成，援疆工作按照计划顺利推进。

　　——切实转变职能，政府自身建设实现新提升。民主法治建设不断深入。制发《鄂州市深化执行力和能力建设，推进以责任为核心的法治政府建设活动的实施方案》，深入推进以责任为核心的法治政府建设，加快构建“职权法定、流程再造、绩效考评、行政问责”为主要架构的依法行政体系。自觉接受人大的法定监督、政协的民主监督和新闻舆论监督，认真办复人大代表和政协委员议案、提案和建议。政务服务水平不断提升。扎实开展“创先争优”和“服务年”活动，坚决纠正部门和行业不正之风。完成市、区（开发区）、街、乡（镇）、村（社区）四级行政服务体系建设任务，市新行政服务中心投入使用，31家行政部门、248项行政审批项目进驻，形成较为完善的城乡一体的行政服务体系。政务公开不断深化，建成了全市统一的电子政务平台，电子监察实现了省、市、区三级联网，市政府门户网站综合考评进入全国前50名，全省政务公开、政务服务和深化预防腐败工作现场会在我市召开。廉洁政府建设不断加强。着力构建惩防并举的腐败风险防控体系，健全和完善反腐倡廉教育、监督、预防、惩治等系列制度，严格规范招投标、土地出让、政府采购、国有资产管理，强化政府投资项目审计，大力实施行政问责和效能监察，不断提高政府工作人员廉洁从政意识。我市被确定为全省“十个全覆盖”示范市、全国廉政风险防控试点市。

　　与此同时，民族宗教、外事侨务、档案方志、新闻出版、对台、保密、供销、工会、老龄、妇女、儿童、侨联、社会科学、机关事务、邮政、通讯、无线电、电力、石油石化等工作都取得了新的成绩。

各位代表，鄂州经济和社会发展的成绩是在极其严峻复杂的挑战中取得的，确实来之不易。倾注了全市各级干部和广大人民群众的心血和汗水，得益于全市上下的团结战斗，各地各部门做了大量艰苦细致的工作，社会各界、各个方面做出了重要贡献。借此机会，我代表市人民政府向辛勤工作在各个岗位上的广大干部群众，向各位人大代表、政协委员，向各民主党派、工商联、无党派人士、人民团体和社会各界人士，向为鄂州发展做出贡献的各位老同志，向解放军指战员、武警官兵以及所有来鄂州的投资者、劳动者和海内外朋友表示崇高的敬意和衷心的感谢！

　　二、“十二五”时期经济社会发展的主要目标和任务

　　“十一五”时期是我市发展极不平凡的五年，也是我市历史上发展最好最快的五年之一，“十一五”规划确定的主要目标全面超额完成。综合实力进一步增强。经济持续快速发展，五年地区生产总值年均增长15%；财政总收入年均增长18.8%；地方一般预算收入年均增长26%；全社会固定资产投资年均增长40.1%，累计增长4倍。经济结构进一步优化。规模以上工业增加值年均增长25.6%，销售收入过亿元工业企业突破100家；农业四大支柱产业规模壮大，水产品产量、加工量居全省第一位；服务业增加值年均增长11.2%，文化教育、社区服务等新兴服务业发展迅猛。承载能力进一步提升。完善主城区和三座新城服务功能，五年共完成城市基础设施建设投资96亿元，完成交通建设投资90.33亿元，均是建市以来投入最多的时期，新增建成区面积23.5平方公里，城镇化率达60.1%，居全省第2位，公路密度居全省第1位,城乡一体化取得突破。改革创新进一步深化。完成市区两级政府机构改革，深化医药卫生体制改革，公立医院改革试点取得重大突破。对外开放水平进一步提高。五年累计外贸出口4.38亿美元，利用外资4.57亿美元，招商引资到位资金356.85亿元。人民生活进一步改善。城镇居民人均可支配收入年均增长12.6%，农民人均纯收入年均增长13.7%，城乡居民人均住房建筑面积达36.8平方米，医疗保险综合参保率达93%，城乡低保保障面达5%以上，人民生活水平逐步向全面小康型转变。鄂州已经进入到一个全新的发展阶段，站在一个更高、更新、更坚实的平台上。

　　五年的发展成就令人鼓舞，但我们必须清醒地看到，鄂州仍处在爬坡上坎、负重前进的发展阶段。当前，还面临许多亟待解决的矛盾和问题。主要是：做大经济总量和调整经济结构难度增加，面临加快发展和优化结构、转变方式的多重任务；财政开源节流压力较大，一方面，受宏观政策影响，财政收入保持较快增长难度加大。另一方面，重点项目建设、民生投入、各项改革等刚性投入增加，财政收支平衡面临较大压力；改善民生和维护社会稳定任务艰巨，处理好社会利益关系的难度增加；政府自身改革和建设有待进一步加强，社会管理和公共服务职能需要进一步强化，等等。解决这些矛盾和问题，是政府今后的艰巨任务，我们将认真面对，统筹谋划，切实加以解决。

　　各位代表，抢抓重要战略机遇期，实现鄂州跨越发展，是“十二五”规划的立足点和出发点。根据市委《关于制定国民经济和社会发展第十二个五年规划的建议》，市政府编制了《鄂州市国民经济和社会发展第十二个五年规划纲要（草案）》，已提交这次大会审议。

　　“十二五”时期我市国民经济和社会发展的指导思想是：高举中国特色社会主义伟大旗帜，以邓小平理论和“三个代表”重要思想为指导，深入贯彻落实科学发展观，全面贯彻落实党的十七大、十七届五中全会、省委九届九次全会、省委工作会议和全省经济工作会议、市委五届十次全会精神，以科学发展为主题，以加快转变经济发展方式为主线，以加速新型工业化、新型城镇化、农业现代化进程为重点，以改革开放创新为动力，以提高城乡人民生活水平为根本出发点和落脚点，深入实施率先融入武汉城市圈、率先实现城乡一体化发展战略，奋力推动鄂州经济社会科学发展、跨越发展，为加快建设“两型”社会示范区、城乡一体化先行区、宜居宜业组群式大城市打下具有决定性意义的基础。“十二五”期间，我市国民经济和社会发展的总体目标是：地区生产总值年均增长13%以上，全社会固定资产投资年均增长20%以上，规模以上工业销售收入年均增长25%以上，地方财政总收入年均增长20%以上，城镇居民人均可支配收入年均增长11%，农村居民人均纯收入年均增长12%，万元生产总值综合能耗、二氧化碳排放量、主要污染物排放总量均控制在省下达目标以内。

　　“十二五”期间经济和社会发展的任务及措施主要是：

　　（一）抢抓战略机遇期，推进跨越发展。一是抢抓“十二五”难得的战略机遇期。“十二五”时期，全球经济格局深度调整，整体复苏上升，世界市场将加速开放，全球产业将加速转移，国际资本将加速流动，为我市承接国际、东部沿海地区资本和产业转移提供了重大的历史性机遇。中央实施扩大内需和中部崛起战略，为推动我市跨越发展提供了政策支撑。全省“两圈一带”战略全面实施，武汉城市圈“两型”社会综改试验区加速建设，湖北长江经济带新一轮开放开发整体联动，为我市充分发挥比较优势提供了广阔的发展平台。我市在全省发展大局中的地位和作用日趋凸显，近年来积蓄的后劲为我市跨越式发展提供了重要保证。二是加快推进工业跨越发展。推进“工业强市”战略，促进工业发展上规模、调结构、建集群、强体系，形成绿色、集约、高端有竞争力的产业体系。大力发展先进制造业。改造传统产业，做大做强冶金、建材、装备制造等支柱产业，壮大提升轻工、纺织服装、农副产品加工、建筑业等优势传统产业。加快发展高新技术产业和战略性新兴产业。突破性发展信息产业、医药化工、新材料、新能源等高新技术产业和战略性新兴产业。促进产业集群发展。三是促进服务业跨越发展。按照建设长江中游区域性物流中转基地、金融创新试验区和建设湖泊生态特色鲜明、吴楚文化彰显、以休闲度假为主体的旅游目的地的发展定位，调整服务业发展思路，由内需带动转向外需带动，推进服务业跨越发展。加快完善服务业布局。突破性发展生产性服务业。积极发展生活性服务业。

　　（二）建设“两型”社会示范区，推进转型发展。积极先行先试，加速武鄂同城化，深化圈域融合，建设城市生态文明，努力建设武汉城市圈“两型”社会示范区。一是突出四个方面试点示范。努力建设改革先行区、创新密集区、“两型”展示区、同城化引领区。二是积极推进四大省级战略。推进青-阳-鄂大循环经济示范区、大梁子湖运动休闲生态旅游度假区、武汉新港鄂州港区建设，推进葛店开发区与东湖高新区对接。三是节约集约利用资源。合理利用与保护土地资源。优化利用矿产资源。科学利用水资源。四是加强生态环境保护。实施水生态修复。狠抓节能减排。实施森林生态保护。建设梁子湖生态屏障保护区。

　　（三）建设城乡一体化先行区，推进统筹发展。以推进“三化同步”（新型工业化、新型城镇化、农业现代化）、“两增同步”（农业增产、农民增收）和“七个一体化”为目标，大力发展现代都市农业，建立农民增收的长效机制，完善城乡基础设施建设，促进城乡公共服务均衡化，探索城乡社会发展新模式，推动城乡经济社会统筹发展。一是大力发展现代都市农业。继续发展特色农业。大力推进农业产业化经营。切实提高农业综合生产能力。大力提高农民收入。二是统筹城乡基础设施建设。建设城乡一体的交通网、信息网、市场网、供水网、供电网、供气网、污水垃圾处理网。三是建设农村新社区。四是推进城乡基本公共服务均衡化。

　　（四）按照新型城镇化要求，推进协调发展。探索特色城镇化道路，合理配置城镇空间资源，提升区域服务功能，改善生态环境，提高城市管理水平，构建功能综合化、环境生态化、资源集约化和生活社区化的新型城市形态。一是优化城市空间布局。提升城镇承载能力和辐射功能。到“十二五”末，建成区面积达到100平方公里，其中主城区建成区面积60平方公里，人口60万，城镇化率达到67%。二是构建配套完善的城镇功能体系。编制完成主城区专业规划。三是科学规划建设城市休闲景观带。充分利用鄂州丰富的景观特色资源，加快形成城市休闲景观结构。四是加强城市基础设施建设。编制全市域基础设施专项规划，加强综合交通体系建设，加强市政基础设施建设，加强能源基础设施建设，加强信息基础设施建设。五是提高城市管理水平。进一步优化城市管理体制机制，加强“数字鄂州”建设，推行“大城管”和“数字化城管”，整体提升城市管理水平。

　　（五）实施科教兴市和人才强市战略，推进创新发展。全面落实鄂州中长期科技、教育、人才规划纲要，推进改革开放，着力提高自主创新能力，切实推进创新型城市建设。一是提高自主创新能力。构建开放型创新体系。完善创新服务体制。推进企业自主创新。加大科技投入。二是优先发展教育事业。加大教育投入，加快发展学前教育，均衡发展义务教育，加快普及高中教育，大力发展职业教育，全面实施素质教育。三是实施人才强市战略。加快培养和引进创新型人才。改善人才发展环境。四是全面推进改革开放。创新行政管理体制。深化经济体制改革。推进综合配套改革。加快发展开放型经济。

　　（六）努力保障和改善民生，推进和谐发展。以建设和谐鄂州为目标，将保障和改善民生作为经济社会发展的根本出发点和落脚点，努力让人民群众共享改革发展成果。一是提高人民生活水平。实施积极的就业政策。提高人民收入水平。探索完善城乡一体的社会保障体系，努力实现养老保险、医疗保险、低保、社会救助的市域全覆盖。扩大城乡居民消费。二是努力提高人民健康水平。做好人口与计划生育工作。切实维护妇女儿童合法权益。完善公共卫生和医疗服务体系。倡导全民体育健身理念。三是建设社会主义精神文明。加强思想道德建设。深化文化体制改革，构建覆盖城乡的公共文化服务体系，加快发展文化产业。四是建设社会主义民主政治。加强民主政治建设。加强法治社会建设。加强反腐倡廉工作。加强国防建设和“双拥”工作。五是建设公共安全管理体系。增强防灾减灾能力。加强应急机制建设。加强食品药品安全监管。提高安全生产水平。切实维护社会稳定。六是推进社会综合管理创新。完善管理架构。创新管理方式。增强管理合力。

　　三、2011年工作安排

　　2011年是“十二五”规划的开局之年，也是鄂州跨越发展的关键之年。全市经济社会发展的主要预期目标为：生产总值增长13.5%，地方一般预算收入增长16%，全社会固定资产投资增长25%，规模以上工业增加值增长23.5%，社会消费品零售总额增长17%，招商引资总额增长35%，实际利用外资增长10%，外贸出口增长12%，城镇居民人均可支配收入增长11%，农村居民人均纯收入增长12%，居民消费价格指数与省保持一致，城镇登记失业率控制在4.5%以内，万元生产总值综合能耗、二氧化碳排放量、主要污染物排放总量完成与省签订的责任目标。今年要重点抓好七个方面的工作。

　　（一）着力转方式，切实推进“两型”社会示范区建设。一是全面推进各项改革创新。修改完善《武汉城市圈“两型”社会建设鄂州示范区改革发展规划纲要》。积极稳妥推进各项改革。加快推进医药卫生体制、户籍制度、土地管理和使用制度、集体林权制度配套、财政支农资金整合和建立多元化投入机制、城乡共治的行政管理体制等改革。完善四级行政服务体系，积极打造高效透明的政务环境，为企业和群众提供优质、便捷的服务。二是积极主动推动圈域融合。加快“五个一体化”和“九个联合体”建设。推进葛店开发区与武汉东湖高新技术开发区全方位融合。加快武汉城市圈葛店职教园建设，建设武汉城市圈职教培训见习基地。组织实施《梁子湖生态环境保护规划》，加快大梁子湖运动休闲生态旅游度假区规划和建设，加快红莲湖新城与梧桐湖新区对接。三是加快青-阳-鄂大循环经济示范区建设。集中人力、物力、财力，形成合力，尽快实施一批建设工程。加快鄂州开发区省级循环经济示范区建设，加快鄂州开发区循环经济示范园、西山循环经济工业园、湖北星丰金属资源利用、拉法基新型建材、皇明太阳能产业基地、天普太阳能产业基地建设，积极申报国家级循环经济示范区。

　　（二）着力调结构，切实提高产业综合竞争力。一是努力提升新型工业化水平。大力改造提升冶金、建材、服装等传统支柱产业，加快鄂钢设备大型化改造等重大技改项目进度，带动支柱产业整体提档升级。发展壮大信息产业、生物医药、新材料、高端装备制造业，突破性发展新能源、节能环保等高新技术产业和战略性新兴产业，抢占新一轮产业发展的战略要地。抓好重点工业项目建设，着力做好鄂钢宽厚板二期、鄂钢大棒、球团二期、程潮铁矿新选矿厂、嘉吉烯王二期、比克电池二期、武钢煤焦油、武汉港工业园等重大项目的建设工作，整体提升工业经济实力。二是加快发展现代服务业。大力发展现代物流、金融保险、信息传输和中介服务等生产性服务业，重点建设三江港区综合交通枢纽工程，着力打造长江中游区域性物流中转基地。大力发展旅游、文化、商贸流通、社区服务和房地产等消费潜力大的生活性服务业，丰富消费服务产品类型。重点做好服务业聚集区培育、服务业重点龙头企业培育、服务业重点项目建设三项工作。重点推进主城区商贸、物流、文化旅游服务业聚集区，葛华科技新城物流、中介服务业聚集区，红莲湖（梧桐湖）运动休闲旅游度假新城服务业聚集区，花湖工贸新城服务业聚集区，梁子湖一湖三地旅游业聚集区等五大聚集区。在每个重点发展领域培育1-2家跨区域、上规模、竞争力强的拥有自主知识产权和知名品牌的服务业龙头企业。三是扩大对外贸易与经济技术合作。继续保持外贸出口持续稳定增长的良好态势。在巩固和发展现有出口企业的同时，不断培植和引进外贸出口企业和项目；优化出口商品结构，扩大高新技术、机电及农产品的出口。鼓励和支持企业对外投资与劳务合作，创新合作方式，扩大合作规模。加大对外人才交流力度，大胆引进优秀人才。进一步拓宽与国外友好城市的交流渠道，开展多领域合作。

　　（三）着力增后劲，切实加快重大项目建设。扎实做好“十二五”规划确定的重大项目的启动工作，为经济发展提供项目支撑。一是强力推进重点项目建设。积极推进“三个一百工程”，加快前期工作，确保顾地总部、中国绿谷太阳能基地、晋煤物流等签约项目和武钢焦化深加工基地、星丰金属资源利用等前期项目开工建设，加快商控华顶、武汉港工业园、汉鄂高速、城际铁路等项目建设进度，确保理想环保数控、银龙管业等竣工项目尽快形成新的生产能力。加大招商引资力度，力争引进一批经济效益好、产业关联度大、技术含量高的项目。二是努力做好重点项目储备争取工作。围绕城乡一体化和“两型”社会示范区、双千亿元产业计划、湖网修复保护和开发利用等重点建设工程，做好重点项目储备，策划一批农产品加工、农业综合能力建设和水利设施建设项目，策划一批绿色、集约、高端产业支撑的工业项目，策划一批生产性物流业、文化产业、旅游业、金融保险业类服务业项目，策划一批城镇交通基础设施项目。对已策划项目不断深化前期工作，加强与省和国家部委沟通衔接，尽可能将我市重点建设项目列入国家和省规划项目库之中。积极做好2011年中央资金项目争取工作，力争取得新的突破。努力做到规划进本子、项目进笼子、资金进盘子。三是不断拓展融资渠道。充分发挥城投公司、交投公司的融资平台作用，做实做强融资平台，增强经营能力。制定好2011年土地储备供应方案和投融资计划。采取BT、BOT等方式，大力吸引民间投资。积极争取银行贷款，努力探索发行企业债券、外国政府贷款、世行亚行贷款等新途径，多方吸引外资和社会资金投入城镇功能设施建设。组建贷款公司，大力引进股份制商业银行，加大农村信用社产权制度改革力度，鼓励企业上市融资。四是强化项目协调服务。继续实行市级领导联系重点项目责任制和派驻工作队帮扶制，进一步明确职责，切实帮助项目建设单位解决征地、拆迁、资金等难题。加强项目督查督办，确保国家省市重点项目的顺利实施。

　　（四）着力促统筹，切实构建城乡一体化新格局。一是打造新城镇。加快特色镇建设，重点支持特色镇完善功能配套，着力打造工业重镇、商贸强镇、旅游文化名镇，积极推进镇级市试点。创新城镇管理体制机制，加快培育小城镇主导产业，提升城镇承载能力和辐射功能。二是建设新社区。建好农村新型社区示范点，积极引导农民向现代生产生活方式转变。加大体制机制创新，积极探索农村治理新模式。结合农村新社区建设，投入4500万元统筹推进农村环境连片整治。三是培育新产业。提高农业综合生产能力，发展壮大水产、畜牧、蔬菜、林果四大支柱产业。大力推进农业产业化，围绕“四个一批”工程培育壮大一批重点龙头企业和一批知名品牌。抓好华容省级农产品加工园建设。加快培育和发展农业专业合作组织，实施农业产业化龙头工程，新增销售收入过亿元企业2-3家。加快发展休闲农业和观光农业，培育农业新的经济增长点。推进农业生产经营方式转变，培育农民企业家。四是配套新设施。统筹城乡水利、交通、信息、市场、供排水、供电、供气等基础设施建设，强力推进城市基础设施向农村延伸。加强农田水利基本建设，财政列支1000万元用于2010年水毁工程的修复重建。抢抓水利改革发展的机遇，积极争取项目和资金大兴农田水利，筹资1.7亿元，开工建设100处农田水利基本建设重点工程。着力做好中小河流整治、湖网生态修复、水库整险加固、内湖堤堰除险保安、涵闸泵站更新改造和抗旱渠道修复等工程建设，确保大灾之后有大治。加大全国农村土地综合整治项目的实施力度。积极推进农户科学储粮减损工程。进一步改善农村生产生活条件，实现城乡空间融合、功能互补、设施共享。五是提供新服务。进一步落实《关于均衡城乡公共服务的意见》30条，以劳动就业、社会保障、基础教育、公共卫生和基本医疗、公共文化、住房保障等为重点，加快推进城乡基本公共服务均衡化，构建城乡政策均等、与经济发展水平、财政保障能力、群众期待相适应的基本公共服务体系。

　　（五）着力强功能，切实提升城市宜居宜业度。今年市政府将多方筹资继续实施基础设施建设“十大工程”，不断完善城市功能。一是不断加大城市建设。筹资8.7亿元，加大城建重点工程投入。加快城市南北中轴线（迎宾大道）南段道路及排水工程、城市东西中轴线西段等续建工程建设。新开工建设迎宾大道南段公铁立交及鄂州南互通、主城区人行天桥等路桥工程。抓好城区道路刷黑、洋澜湖生态修复、绿化景观建设和基础设施维护，推进背街小巷改造。推进火车站广场等园林绿化工程，大力改善人居环境，完善城市综合功能。进一步完善开发区基础设施建设，加快推进鄂城新区建设。二是切实加快交通建设。公路路网方面，确保汉鄂高速、鄂黄高速公路、葛湖公路、316国道、红莲湖高新四路、六路等项目顺利推进。铁路设施方面，加快推进武鄂黄（黄）两条城际铁路、鄂州火车站改造、三江港铁路物流中心及港区铁路工程等项目。港航方面，加快鄂钢三江港矿石件杂货码头、星丰金属资源利用码头、超凡物流码头、五丈港区多用途码头、三和管桩码头建设。三是强力推进“四城联创”。继续做好国家卫生城、环保城、文明城创建工作，力争取得明显成效。做好国家园林城复检工作。深入开展“楚天杯”创建活动。

　　（六）着力惠民生，切实做好社会管理创新工作。一是切实解决民生问题。全面落实税费减免、小额担保贷款、资金补贴等政策，继续实施劳动密集型企业贷款贴息，鼓励全民创业。做好农村劳动力转移培训、高校毕业生就业、困难群众就业工作，保持就业局势基本稳定。

　　大力推进城乡社会保障一体化，不断扩大社会保险覆盖范围。继续提高企业退休人员养老金水平。积极推进华容区、葛店开发区新型农村养老保险试点。大力加强以公共租赁房为主的保障性住房建设，解决住房困难家庭的住房困难问题。继续推进非公经济单位住房公积金制度，努力改善职工住房条件。健全以城乡低保为基础，医疗、教育、住房、临时救助和农村“五保户”、城市“三无对象”、城乡孤儿供养为重点的社会救助体系。二是大力发展社会事业。努力做好社会管理创新试点工作。贯彻中央和省《中长期教育改革和发展规划纲要》精神，进一步落实以区为主的义务教育管理体制。加大教育投入，强力推进义务教育均衡发展。加快发展职业教育，推进鄂州职业大学国家骨干高职院校建设工作，扩大中职招生规模。加快科技进步步伐，提高整合科技资源、组织重大科技活动的能力，完善区域科技创新体系，提升自主创新能力。深化文化体制改革，建成一批基层文体设施，大力开展丰富多彩的文体惠民活动。加快发展广播电视事业。加快市中心医院改扩建步伐，规划兴建市精神卫生中心。建立鄂州市区域卫生信息系统。加强基层医疗服务体系建设，落实乡村医生承担公共卫生服务等任务的补助政策，促进基本公共卫生服务均衡化。加强卫生监督体系建设，卫生监督覆盖面达100%。加强城乡计生服务体系建设，稳定低生育水平，综合整治出生人口性别比偏高问题。大力推进生态文明建设，严格执行环境准入制度，着力强化环保执法，继续抓好污染物减排工作，提高主城区空气质量。重视人口老龄化问题，加快城乡养老服务机构建设，发展社区养老服务事业。推进残疾人事业发展。实现34个城市社区、50个农村新社区服务中心和服务站全覆盖。依法做好村委会换届选举工作。做好新时期的民族宗教工作，推进新一轮援藏援疆工作，启动对口支援民族县市工作。三是全力维护社会稳定。全力做好社会矛盾化解、社会管理创新、公正廉洁执法三项重点工作，加强社会治安防控体系建设，加大打黑除恶力度，完善各级应急预案，增强人民群众的安全感。高度重视安全生产，落实安全生产责任制，建立重大危险源监控网络，加大重点领域安全生产隐患排查整改力度，大力推进以消防队伍为主体的应急救援队伍建设，提高应急救援能力。加强对食品药品安全监管，确保全市人民饮食用药安全，创建食品药品安全先进市。拓宽社情民意的诉求渠道，构建“网络信访、开门接访、主动约访、带案下访、上门回访”的信访工作格局，促进社会和谐稳定。四是继续办好“十件实事”。从群众最关心、最直接、最现实的利益问题入手，集中力量办好加强社会保障工作、实施城镇住房安居工程、扩大就业再就业、提升医疗卫生服务水平、促进教育均衡发展、优化农民生活环境、改善农村生产条件、改善城镇居民生活环境、改善市民出行条件、开展敬老优待服务十件实事。

　　（七）着力优环境，切实加强政府自身建设。加强政府自身建设，营造良好的政务环境，是做好政府各项工作的保证。一是推进依法行政。进一步完善依法行政体系。自觉接受人大及其常委会的法定监督和政协民主监督，充分发挥各民主党派、工商联、无党派人士参政议政作用。认真办理人大代表的建议、批评、意见和政协委员的提案，做到件件有办复、事事有着落。支持工会、共青团、妇联等群团组织参与社会管理和公共服务。扩大基层民主，保障人民依法直接行使民主权利。完善听证、论证和专家咨询制度，建立健全公众有序参与政府决策的制度，提高决策的科学化、民主化水平。二是推进确责履责。在机构改革“三定”工作的基础上，明确行政机关各岗位能力席位标准，规范公务员履责行为，完善行政监督机制，建立健全“确责、履责、问责”的政府责任体系。积极推进建立政府绩效评估体系，将各部门、各级政府履行法定职责、完成工作任务等情况，运用现代管理信息技术手段进行综合评估。积极推行行政问责制，推动工作责任的落实，提高政府的执行力和公信力。三是推进政务公开。继续加强电子政务、市政府门户网站等政务平台建设，健全新闻发言人制度，完善政务、事务、厂务、村务公开，创新政务公开方式，强化公开考核，不断提升行政权力运行的透明度。四是推进能力提升。继续推进政府执行力和以责任为核心的法治政府建设，着力提高领导干部谋划发展、统筹协调、公共服务、社会管理、维护稳定的本领，着力提高公务员的操作能力，把提高执行力和能力建设落实到为经济发展提供良好的政务环境，为市场主体提供优质高效的服务上来。继续深化行政审批制度改革，进一步精简行政审批事项，优化行政审批流程，实行联合审批、集中办理，提高审批效率。五是推进作风转变。大兴密切联系群众之风，大兴求真务实之风，大兴艰苦奋斗之风，坚决克服官僚主义、形式主义。真正把心思放在干事业上，把本领用在促发展上，把功夫下在抓落实上。继续完善市、区、乡镇、村四级行政服务体系和标准化建设，推行集约化的服务模式、精细化的窗口管理，不断提高行政服务效能和质量。六是推进反腐倡廉。严格执行党风廉政建设责任制,加强对主要领导干部经济责任、专项资金和重大投资项目审计监督，健全权力运行制约和监督机制。强化公务员队伍廉洁纪律教育，深入推进“十个全覆盖”，从源头上预防治理腐败。积极开展廉政风险防控，强化制度建设规范政府行为。严肃查处各类违纪违法案件，坚决依法查处损害群众利益的行为。

　　各位代表，鄂州改革开放和现代化建设进入了一个全新的发展阶段。在新的起点上推进鄂州科学发展、跨越发展，是时代赋予我们的光荣使命，是全市人民对我们的殷切期望，是我们必须履行的神圣职责。让我们同心同德，锐意进取，励精图治，真抓实干，实现鄂州更好更快发展，以优异成绩迎接建党90周年！
